# Pilot study using 3D–longitudinal strain computation in a multi-parametric approach for best selecting responders to cardiac resynchronization therapy

**DOI:** 10.1186/s12947-017-0107-6

**Published:** 2017-06-17

**Authors:** Maxime Fournet, Anne Bernard, Sylvestre Marechaux, Elena Galli, Raphael Martins, Philippe Mabo, J. Claude Daubert, Christophe Leclercq, Alfredo Hernandez, Erwan Donal

**Affiliations:** 1 Cardiologie et CIC-IT 1414, Centre Hospitalier Universitaire de Rennes, F-35000 Rennes, France; 2 LTSI, Université Rennes 1, INSERM, F-35000 Rennes, France; 3 Service de Cardiologie, CHU Tours, F-37000 Tours, France; 4 Service de Cardiologie, Saint Philibert Catholic University Hospital, Lille, France; 5 Service de Cardiologie, Hôpital Pontchaillou, CHU Rennes, F-35033 Rennes, France

**Keywords:** Three-dimensional echocardiography, Heart failure, Cardiac resynchronization therapy, Dyssynchrony

## Abstract

**Background:**

Almost all attempts to improve patient selection for cardiac resynchronization therapy (CRT) using echo-derived indices have failed so far. We sought to assess: the performance of homemade software for the automatic quantification of integral 3D regional longitudinal strain curves exploring left ventricular (LV) mechanics and the potential value of this tool to predict CRT response.

**Methods:**

Forty-eight heart failure patients in sinus rhythm, referred for CRT-implantation (mean age: 65 years; LV-ejection fraction: 26%; QRS-duration: 160 milliseconds) were prospectively explored. Thirty-four patients (71%) had positive responses, defined as an LV end-systolic volume decrease ≥15% at 6-months. 3D–longitudinal strain curves were exported for analysis using custom-made algorithms. The integrals of the longitudinal strain signals (I_*L*,peak_) were automatically measured and calculated for all 17 LV-segments.

**Results:**

The standard deviation of longitudinal strain peak (SDI_*L,peak*_) for all 17 LV-segments was greater in CRT responders than non-responders (1.18% s^−1^ [0.96; 1.35] versus 0.83% s^−1^ [0.55; 0.99], *p* = 0.007). The optimal cut-off value of SDI_*L,peak*_ to predict response was 1.037%.s^−1^. In the 18-patients without septal flash, SDI_*L,peak*_ was significantly higher in the CRT-responders.

**Conclusions:**

This new automatic software for analyzing 3D longitudinal strain curves is avoiding previous limitations of imaging techniques for assessing dyssynchrony and then its value will have to be tested in a large group of patients.

## Background

Cardiac resynchronization therapy (CRT) has emerged as a relevant therapeutic intervention for the treatment of chronic heart failure [1–5]. Based on current guidelines, patient selection for this costly therapy relies mainly on heart failure clinical status, ejection fraction (EF), and QRS characteristics (width and morphology). The proportion of non-responders remains relatively high, estimated at up to 30% [6, 7].

Several 2D echocardiographic indices of mechanical dyssynchrony have been proposed to better identify therapy responders, yet the lack of reproducibility and the non-optimal quantification of LV mechanical dyssynchrony (LVMD) achievable with these 2D echocardiographic indices in multicenter trials have cast some doubt on the techniques’ clinical applicability [8–10].

In a further advancement, 3D speckle-tracking echocardiography (STE) has been proposed as an alternative and potentially more accurate method for quantifying LVMD and for identifying patients suitable for CRT [11–13].

These different approaches are typically based on analyzing differences in either myocardial velocity timing, by means of tissue Doppler imaging (TDI), or in myocardial deformation using 2D/3D STE. To describe the complexity of LV mechanics, we believe it is essential to perform a combined assessment of LV dyssynchrony and LV regional contractility using STE, particularly by means of longitudinal strain analysis.

We hypothesized that a new approach, based on automatic quantification of the integrals pertaining to 3D regional longitudinal strain signals, could provide valuable additional information about regional LV mechanics and function prior to any CRT procedure. The aims of this pilot study were to describe LV mechanics using 3D echocardiography integral-derived longitudinal strain parameters in patients eligible for CRT and to test the relevance of this new tool for predicting CRT response.

## Methods

### Study population

48 patients referred for CRT device implantation at two institutions, the Saint Philibert Catholic University Hospital (Lille, France) and the Rennes University Hospital (Rennes, France), were included in the study. Indications for CRT implantation were based on the 2010 ESC guidelines for CRT device use in heart failure [14]. The patients had no previous pacemaker or cardioverter-defibrillator implantation. Patients with a poor acoustic window were excluded (*n* = 5).

Ischemic etiology was defined by a history of previous myocardial infarction or prior coronary revascularization or if a > 75% stenosis was observed in ≥1 of the major epicardial coronary arteries [15]. The NYHA functional class reported was the highest reached by the patient. 12-lead surface electrocardiograms (ECGs) were recorded at 25 and 50 mm/s during intrinsic conduction before CRT-device implantation and then were analyzed by Rennes University’s ECG Core Center. The morphology was classified as either LBBB or non-LBBB (non-specific intraventricular conduction delay) [16]. Only patients with a right bundle branch block were excluded.

The devices were implanted by a standard procedure. The electrophysiologist was blinded with respect to the localization of scar and the main aim during the implantation was to obtain the narrowest QRS at the end of the procedure.

All patients provided informed consent to participate in this study, which was performed in accordance with the principles outlined in the Declaration of Helsinki on research in human subjects (CNIL declaration no. 1620030 V. 0).

### Two-dimensional echocardiography and speckle-tracking echocardiography

Baseline echocardiography was performed prior to CRT implantation (ViVid e9; GE Healthcare, Horten, Norway). Digital, routine, gray-scale, 2D Doppler TDI cineloops were obtained from three consecutive cardiac cycles, along with speckle tracking echocardiographic cineloops from one cardiac cycle, all from the apical view (gray-scale frame rate ≥ 50 Hz; color frame rate > 100 Hz; and 2-, 3-, 4-chamber apical views). Off-line analyses were performed on digitally stored images (BT12-EchoPAC PC; GE Healthcare).

The echocardiography examination was conducted according to the American Society of Echocardiography guidelines [17]. The LV volumes and LV ejection fraction (LVEF) were calculated using the biplane modified Simpson’s rule. All of the measurements were averaged for three cardiac cycles. The LV pre-ejection interval and aortic valve closure values were determined using aortic Doppler profiles. LV global longitudinal strain (LVGLS) was measured off-line using automated functional imaging (AFI). After manual tracing of the endocardial LV border in the 4-, 2-, and 3-chamber views over one frame, the endocardial borders were automatically tracked throughout the cardiac cycle. LVGLS was averaged from all of the analyzable segments in all apical views.

Atrio-ventricular delay was calculated as the ratio between LV filling time and the RR interval. Atrio-ventricular dyssynchrony (AVD) was considered significant when the duration of LV filling time resulted <40% of the RR interval [18]. Interventricular dyssynchrony was defined as a left ventricular pre-ejection interval (LVPEI) >140 ms, with or without an interventricular mechanical delay (IVMD) >40 ms [18]. Intraventricular dyssynchrony was defined by the presence of one of the following: a septal to lateral wall delay by color TDI [19] ≥65 ms [20], or the presence of septal flash (SF). SF was defined as an early septal thickening or thinning within the isovolumetric contraction period, as detected both visually from the gray-scale short axis (SAX) and 4-chamber (4CH) views and from the parasternal long axis, SAX, and 4CH views obtained by M-mode [21].

### Three-dimensional echocardiography and speckle-tracking echocardiography

Baseline 3D–echocardiography was performed in each patient prior to CRT implantation using a commercially available echocardiographic system (ViVid E9; GE Healthcare, Horten, Norway), equipped with a 4 V phased-array matrix transducer. Consecutive 6-beat ECG-gated sub-volumes were acquired from the apical approach, using second-harmonic imaging during end-expiratory apnea, to generate the full-volume data set. We paid particular attention to encompassing the entire LV cavity within the data set, which was digitally stored in a raw-data format and was exported to a separate workstation equipped with the 4D–AutoLVQ package (EchoPAC V.110.1.3, GE Healthcare, Horten, Norway) for off-line analysis of STE LV myocardial longitudinal deformation.

**Fig. 1:**
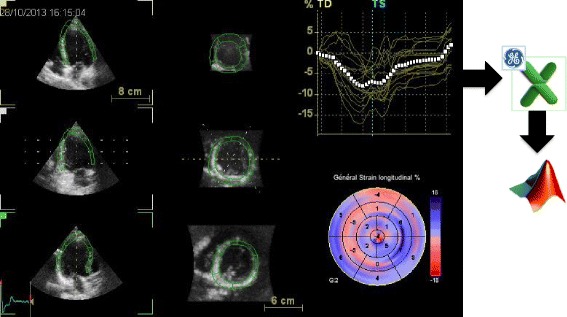
LV dataset display with 3D speckle-tracking analysis of longitudinal myocardial deformation, using the 4D–AutoLVQ package (EchoPAC version 110.1.3, GE Healthcare, Horten, Norway). Microsoft Excel files of 3D longitudinal strain analyses were exported for dedicated analysis performed, with Matlab software (Mathworks Inc., USA)

The end-diastolic frames required for contour detection were automatically displayed in quad view. Manual alignment, achieved by pivoting and translating the four-chamber plane, was undertaken to align the three apical views so that the corresponding intersection line of all of the planes was placed in the middle of the LV cavity, crossing the LV apex and center of the mitral valve in each view. We subsequently used the semi-automated option to identify a fitting geometric model. Importantly, the software required only two single points to be input manually (one at the apex and the other at the tip of the mitral leaflet) on the end-diastolic and end-systolic frames of the four-chamber view slice. The software automatically detected the LV cavity endocardial border in 3D and provided the measured LV volumes. If the endocardial border detection was judged inadequate by the examiner, the LV endocardial borders were manually adjusted in multiplanar layout (three apical and three transverse planes) with a point-click method, immediately followed by secondary automated refinement of boundary detection according to the results. Following assessment of the LV volumes and ejection fraction, an automatic trace of the epicardial border was displayed to identify the region of interest required for LV mass and myocardial deformation measurements by means of 3D STE. This epicardial trace was manually adjusted in order to include the entire LV wall thickness using the same point-click method. The longitudinal deformation parameters were reported as global (both peak and end-systolic) and regional (only end-systolic) and were presented as color-coded polar maps and time-strain traces of an LV 17-segment model (Fig. 1). The time required for all this process is close to the time required to record the acquisitions and the export them on a computer where the computation will be performed automatically.

### Automatic longitudinal strain integrals – new application

**Fig. 2:**
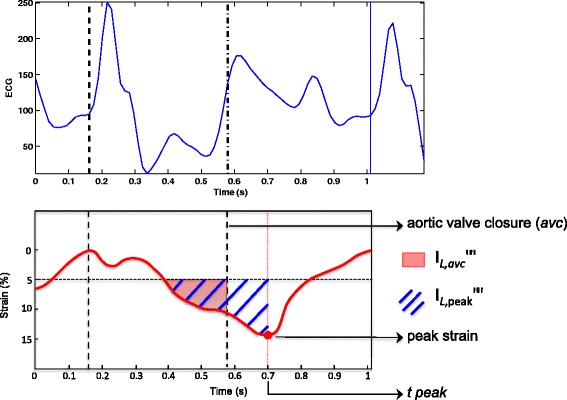
Longitudinal strain curve of one LV segment, analyzed using custom-made algorithms. The *pink area* represents the integral of the longitudinal strain signal from the beginning of the cardiac cycle (QRS onset) to the instant of the aortic valve closure (I_*L,avc*_). The *blue-shaded* area represents the integral of longitudinal strain signal from the beginning of the cardiac cycle to the instant of the corresponding longitudinal strain peak (I_*L*,peak_). All values greater than −5% were considered noise and were thus not considered in calculating integrals. *t peak*: time to strain peak

The 3D longitudinal strain signals were exported in text format to undergo dedicated analysis using custom-made methods and algorithms developed in Matlab software (Mathworks Inc., Natik, Massasuchetts, USA). This analysis included a regional characterization of the longitudinal strain integrals and extended to the 3D case of a set of methods initially developed by our group for 2D strain analysis [22]. The strain integrals represented the accumulated strain during different time intervals of the cardiac cycle. In this study, two particular markers were extracted for each of the 17 LV segments: 1) strain integrals from the beginning of the cardiac cycle (QRS onset) to the instant of the corresponding longitudinal strain peak (*I*_*L,peak*_); and 2), strain integrals from the QRS onset to the instant of aortic valve closure (*I*_*L,avc*_) (Fig. 2). All values exceeding −5% were considered to be noise (irrelevant information) and were thus not considered when calculating the integral.

We tested the integral-based indicators of the regional longitudinal strain signals, all of which were automatically calculated, revealing standard parameters such as “peak strain” (amplitude), “mean strain peak”, and “SD_*t,peak*_” (standard deviation of time to strain peak), along with novel measurements as detailed below.

- mean I_*L,peak*_ and mean I_*L,avc*_ represented the mean of I_*L,peak*_ and I_*L,avc*_ of all 17 LV segments, respectively.
- SDI_*L,peak*_ and SDI_*L,avc*_ were the standard deviation of the integrals of the strain signals I_*L,peak*_ and I_*L,avc*_ of all 17 segments, respectively. SDI_*L,peak*_ and SDI_*L,avc*_ corresponded to the energy dispersion for all 17 segments at the longitudinal strain peak and at the instant of aortic valve closure, respectively.
- DiffInt was calculated as the average of all 17 LV segments in terms of the difference between I_*L,avc*_ and I_*L,peak*_ for each segment. This value could be considered an indicator of the wasted energy developed by the ventricle after aortic valve closure.
- MSDI (maximal difference between strain peak instants) was calculated as the ratio of the time difference between the last and first strain peaks occurring during the cardiac cycle and the duration of the cardiac cycle.

### Echocardiographic response

At 6-months post-implant, all of the patients were reassessed with 2D echocardiography. Response to CRT was defined as a ≥ 15% reduction in LV end-systolic volume, compared with baseline [23, 24].

### Statistical analysis

All of the normally distributed data are displayed as the means and standard deviations, with non-normally distributed data expressed as medians and interquartile ranges (IQRs). Normality was evaluated by the Shapiro-Wilk test. Comparisons between the groups were performed using Student’s t-test or the Welch two-sample t-test, with the Mann–Whitney U test applied for normally and non-abnormally distributed data. Categorical variables, expressed as counts and percentages, were compared using the chi-square test or Fisher’s exact test, as appropriate. A *p*-value <0.05 was considered statistically significant. Sex, non-ischemic etiology, LBBB morphology, QRS ≥ 150 ms, GLS, atrioventricular dyssynchrony, IVMD >40 ms, LVPEI, DTI septo-lateral delay, septal flash, SD_*t,peak*_, SDI_*L,peak*_, DiffInt, and MSDI were covariates entered into the univariate model.

All variables showing a *p* < 0.1 were inserted in the multivariable logistic regression analysis (stepwise entry method). Variables with a *p*-value <0.1 in the multivariate model were considered possible contributors (according to the fact that this is a pilot study). For all of the variables in the multivariate model, the net odds ratio (OR) was reported, along with its 95% confidence interval (CI) and *p*-value. Receiver-operating characteristics curves were individually constructed for dyssynchrony parameters to determine the optimal threshold (closest to the top-left corner), sensitivity, specificity, positive and negative predictive values, and diagnostic accuracy. To explore the value in predicting response to CRT, of the tested 3D–strain parameters over the known variables (i.e. QRS width, or septal flash) the ROC curves were traced and a comparison was done. In addition, and for assessing the complementarity or the concordance of the new potential 3D–strain tool vs the previously proposed predictive parameters, a Kappa test was performed. The analyses were performed using R software, version 3.0.3 (R Foundation for Statistical Computing, Vienna, Austria. URL: http://www.R-project.org/).

## Results

### Study population

A total of 48 heart-failure patients (mean age: 65 ± 10 years, 30 men) referred for CRT device implantation were included in this study. 15 patients (31%) had ischemic cardiomyopathy. The mean intrinsic QRS duration was 160 ms (IQR: 160–170), and a typical LBBB morphology was observed in 39 (79%) patients. The mean LVEF was 26 ± 6%.

At 6-months follow-up, 34 34 patients (71%) had a LV end-systolic volume reduction ≥15% (responders). Compared to non-responders, the responders were more often female (47 versus 14%, *p* = 0.033), had and had better LV performance at baseline as indicated by LVEF (28 ± 5% versus 23 ± 5%, *p* = 0.002), and GLS values (−9.8 ± 3.4% versus 6.5 ± 3.1%, *p* = 0.003). The main clinical and echocardiographic characteristics are shown in Table 1.

**Table 1:** Baseline characteristics of patients

	All patients (*n* = 48)	CRT responders (*n* = 34, 71%)	CRT non-responders (*n* = 14, 29%)	*P* value
Age (years)	65 ± 10	64 ± 10	65 ± 11	0.893
Male, *n* (%)	30 (63%)	18 (53%)	12(86%)	0.033*
Ischemic etiology, *n* (%)	15(31%)	8 (24%)	7 (50%)	0.094
Heart rate (bpm)	69 ± 12	68 ± 12	71 ± 11	0.489
QRS duration (ms)	160 [160; 170]	160 [160; 170]	160 [153; 170]	0.649
QRS & 150 ms, *n* (%)	40 (83%)	29 (85%)	11 (79%)	0.676
LBBB morphology, *n* (%)	38 (79%)	27 (79%)	11 (79%)	1
ACE inhibitors or AR blockers, *n* (%)	46 (96%)	33 (97%)	13(93%)	0.503
β-blockers, *n* (%)	46 (96%)	33 (97%)	13(93%)	0.503
Diuretics, *n* (%)	28 (58%)	16 (47%)	12(86%)	0.014*
Antialdosterone, *n* (%)	18(38%)	14(41%)	4 (29%)	0.412
LVEF (%)	26 ± 6	28 ± 5	23 ± 5	0.002*
LVEDV (ml)	225 ± 85	209 ± 78	265 ± 89	0.037*
LVESV (ml)	169 ± 68	152 ± 57	207 ± 78	0.009*
Mitral regurgitation grade lll-IV, *n* (%)	10(21%)	7(21%)	3(21%)	0.0767
TAPSE (mm)	21 + 4	21 ± 4	20 ± 5	0.78
GLS (%)	− 8.9 ± 3.6	− 9.8 ± 3.4	− 6.5 ± 3.1	0.003*

Data are presented as n (%), mean ± SD, median [IQR]. *ACE* angiotensin-converting enzyme inhibitor, *AR* angiotensin receptor, *GLS* global longitudinal strain, *LVEDV* left ventricular end-diastolic volume, *LVEF* left ventricular ejection fraction, *LVESV* left ventricular end-systolic volume, *TAPSE* tricuspid annular plane systolic excursion. * *P* value <0,05

### Classical dyssynchrony echocardiographic parameters

The classical dyssynchrony parameters were analyzed manually, with the results displayed in Table 2. No significant difference was observed in atrioventricular dyssynchrony between CRT-responders and non-responders. The prevalence of IVMD >40 msec and of septal flash were significantly higher in responders than in non-responders (91 versus 50%, *p* = 0.003 and 79 versus 21%, *p* = 0.001, respectively).

**Table 2:** Classical dyssynchrony 2D–echocardiographic parameters and 3D- echocardiographic integral-based indicators of longitudinal strain

	All patients (*n* = 48)	CRT Responders (*n* = 34, 71%)	CRT Nonresponders (*n* = 14, 29%)	*p* Value
Atrioventricular dyssynchrony, *n* (%)	23 (48%)	16 (47%)	7 (50%)	0.853
IVMD >40 ms, *n* (%)	38 (79%)	31 (91%)	7 (50%)	0.003*
LVPEI (ms)	171 ± 27	175 ± 27	164 ± 28	0.189
DTI septo-lateral delay (ms)	110 [74;161]	114 [74;189]	93 [72;117]	0.162
Septal Flash, n (%)	30 (63%)	27 (79%)	3 (21%)	0.001*
Mean strain peak (%)	−10.2 [−11.6;-9.2]	−10.6 [−11.7;-9.6]	−9.7 [−11.3;-8.4]	0.302
^SD^tpeak ^(ms)^	104 [80;123]	101 [80;123]	107 [66;121]	0.626
^Mean I^L,peak ^(^%.^s-1)^	1.68 ± 0.59	1.80 ± 0.62	1.39 ± 0.41	0.029*
Mean I^avc (%.s^−1^)	0.62 [0.34;0.90]	0.76 [0.44;0.92]	0.45 [0.24;0.77]	0.129
SDIL,peak (%.s^−1^)	1.09 [0.82;1.32]	1.18 [0.96;1.35]	0.83 [0.55;0.99]	0.007*
SDIL,avc (%.s-^1^)	0.85 ± 0.37	0.90 ± 0.35	0.72 ± 0.39	0.125
DiffInt (%.^s-1^)	0.57 ± 0.5	0.61 ± 0.47	0.47 ± 0.58	0.360
MSDI (ms)	0.35 ± 0.16	0.37 ± 0.16	0.29 ± 0.14	0.106

Data are presented as *n* (%), mean ± SD, median [IQR]. *DiffInt* average of 17 LV segments of the difference between I_L_,_avc_ and I_L,peak_ for each 17 LV segments, *DTI* doppler tissue imaging, *LVPEI* left ventricular pre-ejection interval, *I*_*L,avc*_ integrals of longitudinal strain signals for each 17 LV segments from the beginning of the cardiac cycle (QRS onset) to the instant of aortic valve closure, *I*_*L,peak*_ integrals of longitudinal strain signals for each 17 LV segments from the beginning of the cardiac cycle (QRS onset) to the instant of the corresponding longitudinal strain peak, *IVMD* interventricular mechanical delay, *MSDI* Maximal Difference between Strain peak Instants, *SD* standard deviation, *SDI*_*L*_ *,*_*peak*_ standard deviation of the integrals of strain signals I_*L,peak*_ of 17 LV segments, *SDI*_*L,avc*_ standard deviation of the integrals of strain signals I_*L,avc*_ of 17 LV segments, *t*_*peak*_ time to strain peak. * *p* Value <0,05

### Automatic analyses and 3D integral-derived longitudinal strain parameters

In the overall population, the median strain peak was −10.2% (−11.6; −9.2), with no significant difference between CRT responders and non-responders (−10.6% [−11.7;-9.6] versus −9.7% [−11.3;-8.4], *p* = 0.302). The same relationship was observed for SD_*t,peak*_ (101 ms [80; 123] versus 107 ms [66; 121], *p* = 0.626).

In the study population, mean I_*L,avc*_ was lower than mean I_*L,peak*_ (the mean of the differences: 0.97% s^−1^, 95% CI: 0.82–1.13, *p* < 0.0001), and only Mean I_*L,peak*_ was significantly higher in CRT responders than in non-responders (1.80 ± 0.62% s^−1^ versus 1.39 ± 0.41% s^−1^, *p* = 0.029).

The SDI_*L,peak*_ of all 17 LV segments differed significantly between CRT responders and non-responders (1.18% s^−1^ [0.96; 1.35] versus 0.83% s^−1^ [0.55; 0.99], *p* = 0.007). DiffInt and MSDI parameters were comparable between CRT responders and non-responders (Table 2).

### Predictors of echocardiographic response

From all of the clinical, electrocardiographic, and echocardiographic variables entered into the model, the univariate regression analysis identified six variables with a *p*-value <0.1(Table 3). The multivariate regression analyses identified three variables as potentials predictors (Table 3): septal flash (OR: 14.1; 95% CI: 3.08–64.9, *p* = 0.001), SDI_*L,peak*_ (OR: 12.1; 95% CI: 0.81–180, *p* = 0.078), and non-ischemic etiology (OR: 5.33; 95% CI: 0.92–31.1, *p* = 0.063). Among the 18 patients without septal flash, the SDI_*L,peak*_ values were higher in CRT responders than in non-responders (1.12 ± 0.26% s^−1^versus 0.77 ± 0.34% s^−1^, *p* = 0.03) (Table 4).

**Table 3:** Factors associated with good response to cardiac resynchronization therapy (univariate and multivariate regression analyses)

	Univariable OR (95% IC)	*p* Value	Multivariable OR (95% IC)	*P* value
Female	5.33 (1.03–27.5)	0.046*	1.64 (0.01–14.7)	0.657
Non-ischemic etiology	3.25 (0.87–12.1)	0.079*	5.33 (0.92–31.1)	0.063^Ŧ^
LBBB morphology	1.05 (0.23–4.83)	0.948		
QRS > 150 ms	1.58 (0.32–7.76)	0.572		
GLS	1.44 (1.11–1.89)	0.007*	1.22 (0.01–1.77)	0.223
Atrioventricular dyssynchrony	0.89 (0.26–3.09)	0.853		
IVMD >40 ms	10.3 (2.12–50.3)	0.004*	4.35 (0.53–36)	0.172
LVPEI	1.02 (0.99–1.04)	0.189		
DTI septo-lateral delay	1.01 (0.99–1.02)	0.1		
Septal Flash	14.1 (3.08–64.9)	0.001*	14.1 (3.08–64.9)	0.001^Ŧ^
^SD^t,peak	49.1 (−)	0.604		
^SDI^L,peak	18 (1.94–167)	0.011*	12.1 (0.81–180)	0.078^Ŧ^
DiffInt	0.55 (0.15–1.97)	0.354		
MSDI	41.8 (0.42–4200)	0.113		

*DiffInt* average of 17 LV segments of the difference between I_L,avc_ and I_L,peak_ for each 17 LV segments, *DTI* doppler tissue imaging, *GLS* global strain longitudinal, *IVMD* intraventricular mechanical delay, *LbBB* left bundle branch block morphology, *LVPEI* left ventricular pre-ejection interval, *MSDI* Maximal Difference between Strain peak Instants, *SD* standard deviation, *SDI*_*L,peak*_ standard deviation of the integrals of strain signals I_*L,peak*_ of 17 LV segments, *t*_*peak*_ time to strain peak. *All potential factors of positive response to CRT identified from the univariate analyses with a *P* value <0,1 were used in the multivariate logistic regression. ^Ŧ^ Variable with a *P* value <0,1 in the multivariate model were considered to be possible contributors of positive response of CRT

**Table 4:** 3D–echocardiographic integral-based indicators of longitudinal strain in patients without Septal flash

	All patients without Septal flash (*n* = 18)	CRT responders without Septal flash (*n* = 7)	CRT non-responders without Septal flash (*n* = 11)	*P* value
SDI_L,peak_ (%.s^−1^)	0.90 ± 0.35	1.12 ± 0.26	0.77 ± 0.34	0.03*
SDI_*L,peak*_ > 1037%.s^−1^	7 (39%)	5 (71%)	2 (18%)	0,049*

Data are presented as n (%), mean ± SD. *SDI*_*L,peak*_ standard deviation of the integrals of strain signals I_*L,peak*_ of 17 LV segments. * *P* value <0,05

**Fig. 3:**
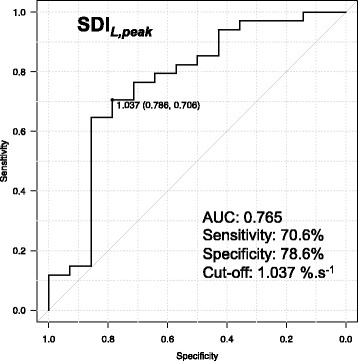
Receiver operator characteristic curve analyses to predict reduction in LVESV ≥15% after CRT for SDI_*L,peak*_

The receiver operator characteristic curve analysis for SDI_*L,peak*_ values identified an optimal cut-off value of 1.037% s^−1^, with a sensitivity (Se) of 70.6% and specificity (Sp) of 78.6% (Fig. 3). The positive predictive value (PPV), negative predictive value (NPV), and diagnostic accuracy for a cut-off of 1.037% s^−1^ were 89%, 52%, and 0.73, respectively (Table 5). For the septal flash, the Se, Sp, PPV, NPV, and diagnostic accuracy were 79%, 79%, 90%, 61%, and 0.79, respectively.

**Table 5:** Sensitivity, specificity, positive predictive predictive value, negative predictive value, diagnostic accuracy in monoparametric and multiparametric analyses for reverse remodeling induced by cardiac resynchronization therapy

	Sensitivity	Specificity	Positive predictive value	Negative predictive value	Diagnostic accuracy
septal flash	79%	79%	90%	61%	0.79
SDI _L,peak_ > 1.037%.s^−1^	70.6%	7.6%	88.9%	52.4%	0.73
SDI _L,peak_ > 1.037%.s^−1^ S Septal Flash	55.9%	92.9%	95%	46.4%	0.67
SDI _L,peak_ > 1.037%.s^−1^ + AV	29.4%	92.9%	90.9%	35.1%	0.48
SDI _L,peak_ > 1.037%.s^−1^ + IV	67.6%	92.9%	95.8%	54.2%	0.75
SDI _L,peak_ > 1.037%.s^−1^ + AV + IV	29.4%	92.9%	90.9%	35.1%	0.48
SDI _L,peak_ > 1.037%.s^−1^ + AV + IV + Septal Flash	23.5%	100%	100%	35%	0.46

*AV* atrioventricular dyssynchrony, *IV* interventricular dyssynchrony, *SDI L,peak* standard deviation of the integrals of strain signals

All of the multiparametric associations were tested, and they are displayed in Table 5. Better diagnostic accuracy was achieved with a combination of an SDI_*L,peak*_ value >1.037% s^−1^ and interventricular dyssynchrony. The association of four parameters (SDI_*L,peak*_ > 1.037% s^−1^, atrioventricular dyssynchrony, interventricular dyssynchrony, and septal flash) increased the test specificity to 100%, although it decreased its sensitivity and NPV. The Kappa test testing the concordance between septal flash and SDI_*L,peak*_ was 0.26 confirming that SDI_*L,peak*_ is exploring another kind of mechanical dyssynchrony than septal flash. In addition combining the septal flash and the SDI_*L,peak*_ > 1.037% s^−1^ lead to an area under the curve: AUC = 0.86.

## Discussion

Our study investigated a new quantitative computation method for longitudinal strain curves. Integral-derived parameters were generated and described for the first time, and they were applied in heart failure patients eligible for CRT, producing the following primary findings: 1) automatic analysis of longitudinal strain curves provided new complementary data on LV mechanics by combining information on timing and LV regional performance; 2) the mean I_*L,peak*_ and SDI_*L,peak*_ of all 17 LV segments were higher in CRT responders than in non-responders; 3) While marginally significant in this study**,** SDI_*L,peak*_ could have an independent value for predicting CRT response; and 4) combining a SDI_*L,peak*_ value >1.037% s^−1^ and interventricular dyssynchrony appeared to be a promising multi-parametric approach for best predicting CRT response, and it was feasible and robust in patients with sufficient acoustic windows and a sinus rhythm. And perhaps the most important finding was that 3D strain-derived parameter could differentiate responders from non-responders among 18 patients without septal flash.

### Automatic versus mechanical manual dyssynchrony analysis

Since the publication of the disappointing PROSPECT study results [8], several questions have remained unanswered regarding the real discriminatory value of mechanical dyssynchrony in CRT candidates.

The first source of error is undoubtedly related to the lack of standardized data-processing methods. Numerous parameters have been proposed [10]. After disappointing results and thanks to effort of scientists, it has been best understood how complex is cardiac mechanical, how complex is electromechanical coupling but also how valuable could be the imaging approach to best select patients for CRT [25–27]. It has been for instance, clearly shown that, patients with the same left-bundle branch block could exhibit completely different mechanical dyssynchrony patterns [28]. But, there are also, technical challenges to consider. The analysis of strain peaks can be difficult because there are different patterns of strain, which often have multiple peaks. Determining the relevant peak or peaks, along with the reproducibility of the timing of these peaks, remains a challenge. To overcome this difficulty and also to consider not only peaks and dyssynchrony but regional myocardial function as well, a number of authors have proposed novel operator-independent methods that automatically assess function and dyssynchrony, using predefined algorithms [28–31]. In our study, heart failure patients with dilated or ischemic cardiomyopathy were considered eligible for CRT. All of the patients presented with severe LV dysfunction, an altered LVGLS as detectable on 2D echocardiography, an altered mean strain peak with a very low amplitude of strain curves, and significant dispersion of time to strain peak (SD_*t,peak*_). We thus proposed a new 3D automatic assessment of regional LV mechanics, avoiding as much as possible potential human error.

### Toward a new step by step approach for assessing left ventricular dyssynchrony assessment and for CRT response prediction

Mechanical dyssynchrony assessment could have 2 steps. As the first step, septal flash should be searched for using a simple visual and/or M-mode approach. If septal flash (and/or apical rocking) is not found, 3D echo should be performed in the next step as an attempt to detect a novel predictor of volumetric response. In the present study, we confirmed such results but also that the septal flash is highly relevant [21]. That is emphasizing the potential value of multiparametric scores like the L2ANDS2 score [32]. However, septal flash was found in 79% of CRT responders and in only approximately 60% of all of the patients, regardless of cardiomyopathy etiology. In patients without any septal flash, SDI_*L,peak*_ was significantly higher in CRT responders, suggesting that this new predictor could provide additional information for the optimal selection of CRT patients. After the performing of a multiparametric evaluation of mechanical dyssynchrony that considers the three levels of dyssynchrony (atrio-ventricular, interventricular, and intra-ventricular), it appeared rather clear that this imaging approach could be of additive value to ECG [33]. A combination of very simple dyssynchrony parameters, such as atrioventricular, interventricular and septal flash, which are easy to measure and are reproducible, with automated strain-derived parameters, should probably be tested in larger groups of patients based on the current, first validation. The combination of an SDI_*L,peak*_ > 1.037% s^−1^ and interventricular dyssynchrony appeared to be an interesting multiparametric approach for predicting a good CRT response.

This “second step” three-dimensional STE appeared promising for several reasons [34]: (I) all 17 segments of the LV were evaluated in their 3D motion, along with the relationships among them, thus avoiding the ‘out-of-plane’ phenomenon inherent to 2D imaging; (II) the full LV volume was assessed during a 6-beat acquisition, allowing for a rapid evaluation of the global and regional all-directional contractions; and (III) all of the 3D echocardiographic strain markers (longitudinal, radial, and circumferential area) exhibited good reproducibility [35]. In our study, the feasibility rate of 3D STE was 83%, with the scientific literature reporting feasibility rates ranging from 63 to 83% [36]. This feasibility rate is likely to increase further with the advent of new transducer technologies. Until now, the principal 3D echocardiography parameter has been the standard deviation of time to minimal systolic volume [11]. This parameter has proved a feasible and reliable parameter of LV mechanical dyssynchrony, which might even provide additional value compared to the current selection criteria for accurate CRT response prediction [11]. This approach is only looking at the analysis of the differences in timings. The assessment of remaining LV regional contractility is lacking [11]. In a small study, 3D strain dyssynchrony index, (area tracking approach using the average difference between peak and end-systolic area strain, derived from 16 LV segments), was proposed for predicting CRT-response [13]. An integrative approach of dyssynchrony and function, using a new longitudinal strain integral-derived method, has appeared more relevant [29, 37]. This approach is likely to be of particular interest in ischemic diseases for distinguishing the passive movement of scarred tissue segments [38]. The mean I_*L,avc*_ was lower than the mean I_*L,peak,*_ in our patients, indicating that most segments reached their maximal deformation after aortic valve closure. SDI_*L,peak*_, corresponding to the energy dispersion for all 17 segments at the longitudinal strain peak, appeared to be promising for the assessment of LV dyssynchrony and the prediction of LV reverse remodeling following CRT.

### Study limitations

That is a first pilot study, using one kind of echo-machine in a limited number of patients but in two centers. The goal was to ‘validate’ a tool that was planned to be use in largest population. Since a large intervendor variability has been demonstrated in 3D strain systems, the cut-off values identified are valid only for GE technology [39]. Only the integral-derived longitudinal strain parameters were measured in this study [40]. One limitation to the image acquisition for 3D speckle-tracking was the relatively slow volume rate of 32 ± 7 volumes/s. The volume rate and the image definition will improved in next generation of 3D–probes, it will improve the value of our automatic approach that will be applicable in atrial fibrillation patients thanks to the single beat capabilities.

Another limitation is related to fact that it is a pilot study with the use of an endpoint that is questionable. LV-remodeling is not a perfect surrogate marker of the hardest clinical endpoints. The Endpoint proposed by Packer will have to be considered [41].

## Conclusion

This new automatic analysis of 3D longitudinal strain curves, using integral-derived parameters, provided original information on LV mechanics by combining timings and LV regional contractility data. This approach could be of value for improving patient selection for CRT.

## Abbreviation

3D: Three-dimensional
4CH: Four-chamber
AFI: Automated function imaging
CRT: Cardiac resynchronization therapy
DiffInt: The average of 17 LV segments of the difference between I_*L,avc*_ and I_*L,peak*_ for each segment
I_*L,avc*_: The integrals of longitudinal strain signals for all 17 LV segments from the beginning of the cardiac cycle (QRS onset) to the moment of the aortic valve closure
I_*L*,peak_: The integrals of longitudinal strain signals for all 17 LV segments from the beginning of the cardiac cycle (QRS onset) to the moment of the corresponding longitudinal strain peak
IQR: Interquartile range
IVMD: Interventricular mechanical delay
LBBB: Left bundle branch block
LV: Left ventricular
LVEF: Left ventricular ejection fraction
LVGLS: Left ventricular global longitudinal strain
LVPEI: Left ventricular pre-ejection interval
MSDI: Maximal difference between strain peak instants
NYHA: New York Heart Association
PPV: Positive predictive value
SD_*t,peak*_: Standard deviation of time to strain peak
STE: Speckle-tracking echocardiography
TDI: Tissue doppler imaging
